# Understanding the Impact of Multiple Sclerosis on Quality of Life: An Italian Pilot Study

**DOI:** 10.3390/brainsci15090960

**Published:** 2025-09-03

**Authors:** Elsa Vitale, Roberto Lupo, Ludovica Panzanaro, Rebecca Visconti, Maria Rosaria Tumolo, Paolo Caldararo, Federico Cucci, Donato Cascio, Giorgio De Nunzio, Stefano Botti, Ivan Rubbi, Luana Conte

**Affiliations:** 1 Directorate of Health Professions and Nursing, Local Health Authority (ASL) of Bari, 70123 Bari, Italy; vitaleelsa00@gmail.com; 2 Department of Surgery, ‘San Giuseppe da Copertino’ Hospital, Local Health Authority (ASL) of Lecce, 73043 Copertino, Italy; roberto.lupo@asl.lecce.it; 3 Comunità Riabilitativa Assistenziale Psichiatrica “CARRUBO”, Sol Levante Srl, 74020 Avetrana, Italy; ludovica.panzanaro@hotmail.it; 4 Independent Researcher, 73100 Lecce, Italy; rebecca.visconti01@gmail.com; 5 Biological and Environmental Sciences and Technology Department, University of Salento, 73100 Lecce, Italy; mariarosaria.tumolo@unisalento.it; 6 Institute of Clinical Physiology, National Research Council, 73100 Lecce, Italy; 7 Gruppo Villa Maria, Città di Lecce Hospital, 73100 Lecce, Italy; paolo.caldararo99@gmail.com (P.C.); fcucci@gvmnet.it (F.C.); 8 Department of Physics and Chemistry “E. Segrè,” University of Palermo, 90128 Palermo, Italy; donato.cascio@unipa.it; 9 Laboratory of Biomedical Physics and Environment, Department of Mathematics and Physics “E. De Giorgi,” University of Salento, 73100 Lecce, Italy; giorgio.denunzio@unisalento.it; 10 Advanced Data Analysis in Medicine (ADAM), Laboratory of Interdisciplinary Research Applied to Medicine (DReAM), Local Health Authority (ASL) of Lecce, 73100 Lecce, Italy; 11 Hematology Unit, Azienda USL-IRCCS of Reggio Emilia, 42100 Reggio Emilia, Italy; stefano.botti@ausl.re.it; 12 Department of Medical and Surgical Sciences, School of Nursing, University of Bologna, 48018 Faenza, Italy; ivan.rubbi@auslromagna.it

**Keywords:** multiple sclerosis, disability, care, coping, inclusion

## Abstract

Backgorund. Multiple sclerosis (MS) profoundly affects the lives of patients and their families. The experience of the disease is shaped not only by its progression and specific characteristics but also by the quality of medical and caregiving support received. The diagnosis of MS represents a transformative event that may lead to job loss, the need for continuous care, and a significant reorganization of family roles. In Italy, more than 140,000 people are affected by MS (AISM data, 2024). The impact of the disease is multifaceted and complex, involving various aspects of the patient’s life. Dependence on external assistance often becomes an unavoidable necessity, highlighting the importance of exploring the quality of life of people with MS in the Italian context. The main objective is to assess the quality of life of individuals affected by MS, both before diagnosis and during the course of the disease. A secondary aim is to identify related psycho-physical consequences, including care-related needs. Methods: An online survey was conducted through various associations operating across Italy, involving a sample of 99 individuals diagnosed with MS. Results: The results show a predominance of female participants, with a mean age of 41 years. The disease was reported to be at an early stage in 66.7% of cases and advanced in 33.3%, with none of the respondents being in a terminal phase. The most frequent clinical form was relapsing–remitting MS (RRMS), which accounted for 78.8% of the cases. In terms of employment and daily activities, more than half of the participants reported underperforming (59.6%) or limiting specific tasks (51.5%) due to disability caused by the disease. Emotional distress had even more pronounced effects, with 63.6% reporting a decline in performance and 62.6% experiencing concentration difficulties. Quality of life was significantly affected, particularly in the physical and emotional domains. Vitality, physical pain, perceived health, and psychological well-being emerged as compromised dimensions, pointing to the need for a multidimensional care model that integrates therapeutic, rehabilitative, and psychosocial interventions. Individuals in the early stages of MS tended to maintain better work relationships and demonstrated higher levels of professional engagement. Conclusions: The findings underscore the importance of a continuous and personalized care approach, addressing not only clinical treatment but also psychological and social support. These aspects are crucial for monitoring patients’ needs, promoting quality of life, facilitating disease acceptance, and mitigating psychological distress.

## 1. Introduction

Multiple sclerosis (MS) is one of the most widespread neurological disorders globally [1]. It is a chronic inflammatory disease of the central nervous system with a broad spectrum of clinical manifestations. MS leads to demyelination and neurodegeneration processes, progressively impairing motor, sensory, and cognitive functions. Its pathogenesis involves genetic, environmental, and immunological factors [2]. In Italy, more than 140,000 people live with MS, with approximately 3600 new diagnoses each year. The disease predominantly affects young women, especially those between the ages of 20 and 40 [3]. According to data from the Italian Multiple Sclerosis Association (AISM, 2024), MS can even, in about 6.5% of cases, begin during school age, negatively impacting educational trajectories and often leading to school dropout and a high risk of stigmatization, as highlighted in a review of the literature [4,5]. This phenomenon is further confirmed by studies on pediatric MS, which show specific clinical features and long-term consequences [6]. Despite recent advances in pharmacological treatments and rehabilitation strategies, caring for people with MS remains a multidisciplinary challenge [7]. As shown in Savio et al.’s study [8], MS requires an integrated approach that includes medical therapies, psychological support, and coping strategies aimed at improving patients’ quality of life. The complexity of MS calls for not only symptom management but also continuous support tailored to enhance patients’ overall well-being. For example, in relapsing–remitting MS patients, treatment with dimethyl fumarate has been shown to improve sleep quality and depression, highlighting the potential benefits of targeted therapies [9].

To date, few studies have provided a detailed account of the quality of life and the associated psycho-physical consequences experienced by individuals with MS within the Italian context. For many people, receiving a diagnosis of MS represents a life-altering event that can profoundly reshape their perception of both the present and the future. A crucial factor in this phase is the sense of self-efficacy, the belief in one’s ability to positively influence their condition through deliberate choices and behaviors. As shown in an Italian study, psychological aspects such as self-esteem and self-efficacy are closely linked to quality of life and are often critical issues for people living with MS [10]. This perception varies between individuals and may shift across different life stages and personal domains. The limited number of studies investigating these dimensions in the literature underscores the need for the present study. The primary objective of this study is to assess the quality of life and the psychological impact experienced by patients with MS, both before and after diagnosis. A secondary aim is to identify the related psycho-physical consequences, including care and assistance needs. These needs inevitably extend to caregivers, who often face fatigue and loneliness, which in turn affect their quality of life. Supportive policies are essential to help caregivers prevent burnout and preserve the stability of home care [11].

## 2. Material and Methods

### 2.1. Study Design

This was a pilot, cross-sectional study.

### 2.2. Study Procedure

The questionnaire was spread throughout Facebook and Istagram social pages in which participants were all SM affected.

In Italy there were several Facebook and social pages embracing MS patients, however the only 2 groups which decided to participate and thus spread the questionnaire on their pages were “Fermiamo la sclerosi multipla” and “Io vivo con la sclerosi multipla,” both of which serve as platforms where individuals with MS share their personal experiences.

The questionnaire was publicized thanks to an active online link from November 2024 to April 2025. In this period participants were free to answer the questionnaire.

### 2.3. Data Collection Tool

Data were collected through the completion of an anonymous online questionnaire, specifically designed for this study and composed of multiple-choice questions.

The questionnaire was divided into five main sections, as follows:

a. Demographic and socio-economic data, aimed at describing the participants’ profiles.

b. Symptoms and clinical history of MS, including information on diagnosis; disease progression, like “early phase” and “advanced phase;” whether the MS diagnosis has been performed before or after 1 year; symptoms before and after diagnosis; and pharmacological treatments.

c. Lifestyle and behavioral habits (smoking, physical activity, previous infections).

d. Care support received and access to professional figures (e.g., neurologist, nurse, physiotherapist).

e. Impact of the disease on quality of life, including emotional, relational, and sexual domains, as well as perceived daily difficulties.

### 2.4. Ethical Considerations

This study complied with all ethical and legal regulations concerning privacy and the protection of personal data. Participants accessed the questionnaire via a digital link after reading and accepting the informed consent form, in compliance with the European General Data Protection Regulation. Full anonymity was guaranteed, and no sensitive or personally identifiable data were collected.

Participants were clearly informed about the purpose and implications of the study, and informed consent was obtained prior to voluntary participation. The study was approved by the ethics committee of Bari-Policlinico (protocol no. 7766/2023).

### 2.5. Statistical Analysis

Data were collected in an Excel spreadsheet, and frequencies and percentages were calculated for all categorical variables in the questionnaire. To assess in detail the impact of disease stage on different dimensions of participants’ lives, the Chi-square test was used. All *p*-values less than 0.05 were considered statistically significant. Statistical analysis was performed using the software SPSS (version n.29).

## 3. Results

### 3.1. Socio-Demographic Characteristics of the Sample

A total of 99 individuals diagnosed with MS participated in the study. Regarding socio-demographic data (Table 1), a clear predominance of female participants was observed, with a mean age of 41 years. The disease stage was reported as early in 66.7% of respondents, while none of the participants were in a terminal phase. The most prevalent clinical form among participants was relapsing–remitting MS (RRMS), accounting for 78.8% of cases.

It is noteworthy that 75.8% of respondents reported no family history of MS, suggesting a possible area for further investigation regarding the role of familial predisposition as in line with the study of Harirchian et al. [12].

The female gender was the majority (n = 79), and 60.6% of participants were currently employed, while 13.1% were unemployed. Concerning the age of disease onset, the 20–29 age group was the most represented. However, both early-onset cases (≤19 years) and late-onset cases (≥50 years) were also reported, highlighting the unpredictable nature of the disease.

**Figure 1:**
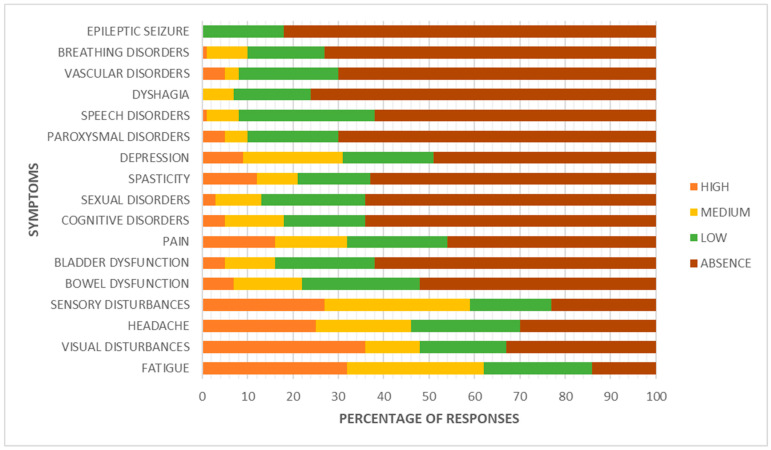
Signs and symptoms.

Fatigue, sensory disturbances, headache, and visual impairments represent early warning signs that are often underestimated, yet commonly present at the onset of MS. More specific and advanced symptoms, such as dysphagia, epileptic seizures, paroxysmal disturbances, or speech disorders, tend to appear later in the disease’s course and are often absent in the initial phase (Figure 1).

Among the most frequently reported consequences following disease onset, fatigue stands out as the most common symptom [13], with 77.8% of patients reporting its presence. Although it is often a subjective and difficult-to-quantify symptom, in 10.1% of cases it led to such a worsening of condition that hospitalization was required, underscoring its potential impact on quality of life. Sensory disturbances are also very common (50.5%), and notably, in 22.2% of cases, they resulted in hospitalization.

Neuropathic pain was reported by approximately one third of participants (34.3%), with a hospitalization rate of 9.1%. This highlights the clinical relevance of chronic pain in MS, which may require specialist intervention during acute phases. Bowel disturbances (39.4%) and urinary symptoms (26.3%) were reported by a non-negligible portion of the sample, though both rarely resulted in hospitalization (3% each).

Spasticity affected 27.3% of patients, and also showed a noteworthy clinical impact, with a hospitalization rate of 10.1%, often linked to functional deterioration or persistent muscle pain. A particularly relevant finding concerns postural tremor associated with recurrent falls: although this symptom was relatively uncommon (17.2%), every patient who experienced it required hospitalization.

**Figure 2:**
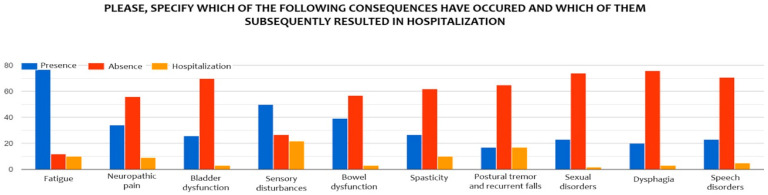
Psycho-physical disorders in the sample.

Sexual and communication disorders were reported at similar rates (23.2%). Finally, swallowing difficulties, present in 20.2% of cases, led to hospitalization in 3% of the sample, indicating that even less frequent symptoms can have significant clinical implications (Figure 2).

### 3.2. Perceived Quality of Life

The analysis of results highlights how the perception of health status among individuals with MS can be highly subjective. Data on perceived health status reveal a heterogeneous distribution. Only a small minority considers themselves in excellent health (2% excellent, 16.2% very good), while the majority fall into an intermediate category: 34.3% rate their health as good and 32.3% as fair. Notably, 15.2% perceive their health as poor, underscoring the significant impact MS can have on overall quality of life and perceived well-being.

The responses collected indicate that MS has a considerable impact on both the physical and emotional dimensions of daily life. About a quarter of respondents reported marked limitations in performing moderately intense physical activities, such as moving a table or climbing stairs, while an even larger proportion (over 30%) reported partial limitations. However, nearly half of the sample reported no limitations in these activities, suggesting a wide variability in perceived disability.

In terms of work and daily tasks, more than half of the respondents stated they performed below their expectations (59.6%) or had to limit certain activities (51.5%). The emotional burden appears even more significant: 63.6% reported a decline in performance, and 62.6% experienced difficulties in concentration.

Currently, 77.8% of participants are undergoing pharmacological treatment. The most commonly used medications include Natalizumab (11%), Teriflunomide (10.1%), Interferon beta-1a and 1b (12.1%), Dimethyl fumarate (9.1%), and Ocrelizumab (8.1%).

Data regarding home-based care support for individuals with MS reveal a significant shortage of adequate services. Only 20.2% of respondents reported receiving adequate home assistance, while the vast majority (79.8%) had no access to such support.

With regard to healthcare professionals involved in patient care, most respondents were followed by a neurologist (72.4%), a key figure in the management of MS. A smaller proportion received support from psychologists (19%) and nurses (12.1%), who are essential in addressing the emotional and practical aspects of the disease. Even fewer participants reported receiving care from physiotherapists (6.9%) or speech therapists (3.4%), suggesting that these services may not be sufficiently accessible or integrated into the care pathway.

A striking 91.9% of participants reported having experienced moments of emotional distress (Table 2).

### 3.3. Forms of Support Received

**Figure 3:**
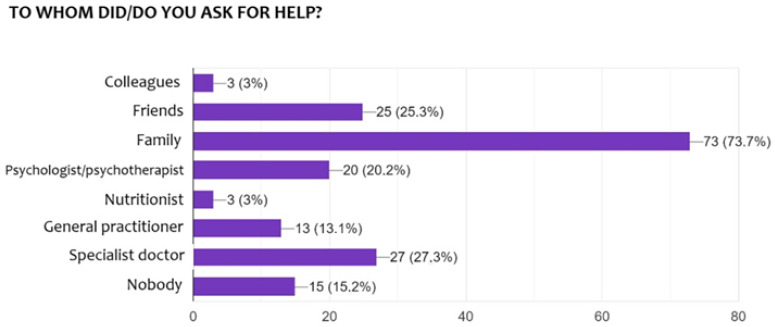
Forms of support received.

In terms of support, family emerges as the primary source of help, with 73.7% of respondents turning to their loved ones during difficult times. To a lesser extent, friends (25.3%) and work colleagues (3%) were also mentioned as sources of support, although their involvement appeared to be more limited. Professional support appears somewhat more prevalent, with 20.2% of participants seeking help from a psychologist or psychotherapist. This confirms previous evidence that social support has a direct and measurable effect on the quality of life of people with MS [14]. A similar proportion reported consulting medical specialists (27.3%) or their general practitioner (13.1%). Finally, 15.2% of respondents stated that they do not seek help from anyone, a figure that may reflect difficulties in expressing emotional suffering or in recognizing the need for external support (Figure 3).

### 3.4. Impact of Disease Stage on Different Dimensions of Life

A detailed statistical analysis was conducted to evaluate the impact of disease stage on various aspects of individuals’ lives (Table 3). The findings reveal that individuals in the early stage of MS tend to maintain better quality work relationships and demonstrate greater engagement in professional activities. Conversely, those in the advanced stage report a greater sense of social isolation and increased difficulties in managing daily tasks, such as catheter use.

The impact of the disease on sexual functioning also emerged as particularly relevant. Patients in the advanced stage reported issues such as loss of desire, reduced lubrication, and inhibitions often linked to sociocultural stigma. These findings reflect the deep influence of MS on personal identity and self-esteem according to Timkova et al.’s study [15].

## 4. Discussion

The objective of this study was to assess the quality of life in individuals with MS. The analysis of the data reveals that patients are predominantly young, an aspect documented in only a few studies in the existing literature [16]. Significant differences emerged in the perception of disease impact depending on the stage of progression. The information gathered allows for a comprehensive overview of the daily challenges faced and the broad influence of the disease on quality of life, both physically and psychosocially.

One of the earliest difficulties appears to arise in the work environment, as supported by findings from an Italian study in which workplace conditions, social relationships at work, negative work events, and lack of information were identified as predictive factors for social marginalization [17]. Psychological adaptation and social reintegration are as essential as appropriate pharmacological treatment and multidisciplinary care. A thorough biopsychosocial assessment is therefore required to address risks and promote protective factors that can improve the quality of life in patients with MS within general medical practice [18]. The management of individuals with MS encompasses both the diagnostic phase and the clinical course, requiring a multidisciplinary approach [19]. This approach should begin at the onset of symptoms and continue throughout the progression of the disease, especially during advanced stages, which are often more burdensome and complex. These stages are marked by a heightened perception of social isolation, uncertainty about the future, and difficulties in maintaining personal autonomy, as evidenced by challenges in catheter management and the lack of institutional support reported in our study. In recent years, the use of MRI in the diagnosis of MS has significantly advanced. The 2017 McDonald criteria enable earlier and more accurate diagnosis but require expert application after excluding alternative conditions. New MRI markers, such as the central vein sign and paramagnetic rim lesions, may improve diagnostic specificity, though further validation is needed [20].

The impact of MS extends beyond the individual and affects families as well [21], particularly in the presence of comorbidities. As shown in an Italian study, 53.8% of individuals with MS presented at least one comorbidity, including hypertension, depression, and anxiety [22]. Of particular concern is the disease’s influence on sexual and relational domains. In everyday clinical practice, sexual dysfunction is often underestimated, as physicians tend to focus on classical neurological deficits and may overlook symptoms that seriously compromise quality of life. These conditions suggest not only a deterioration of physical function but also a profound disruption of body image and self-esteem—factors that can hinder the ability to maintain fulfilling intimate relationships [23,24]. Approximately 30–80% of individuals with MS experience behavioral changes associated with disease progression [25]. These are frequently linked to depression and other neuropsychiatric disorders, and often not associated with motor impairments, suggesting the involvement of distinct pathological mechanisms, as already indicated in an Italian study [26]. Such alterations further contribute to disability in MS [27]; however, both preventive exercise and physical rehabilitation may enhance the expression of LTP-like synaptic plasticity in MS, potentially reducing disability accumulation [20]. In light of this evidence, the importance of an integrated, patient-centered care model becomes clear, one that addresses psychological and social dimensions alongside medical management [28,29]. Personalized rehabilitation programs, combined with ongoing psychological support, are essential tools to preserve quality of life, even in advanced stages of the disease. Neuropsychiatric symptoms and cognitive impairment are often overlooked, and current treatments show limited effectiveness due to complex underlying mechanisms. Improved understanding and evaluation are needed to guide more effective, personalized therapies [30]. This need became even more evident during the COVID-19 pandemic, as a meta-analysis found that people with MS experienced greater psychological distress compared to pre-pandemic levels [11].

The findings of this study confirm the heterogeneity of the subjective experience of MS and highlight the urgency of a multidimensional care model, one that goes beyond clinical symptom management to embrace relational care, social support, and the promotion of patient autonomy throughout the disease trajectory [31].

Within this framework, the nursing role proves to be fundamental [32,33], as it involves coordinating care, monitoring disease progression, and ensuring effective communication between the patient, family, and other healthcare providers.

## 5. Study Limitations

Among the study’s limitations are the online data collection method and the relatively small sample size, which may have affected the representativeness of the findings. In Italy there are about 140,000 MS patients [34]. Thus, more participants could be enrolled. However, we conducted a pilot study and we also recognized that data collection of this manner is challenging for participants. Despite the data collected providing valuable insights, particularly in the context of early diagnosis and person-centered care management, future studies will be performed to reach more participants to achieve a representative sample size.

## 6. Conclusions

The results of this study demonstrate that the quality of life of Italian individuals living with MS is particularly compromised in the physical and emotional domains. In this context, patient engagement and integrated care pathways play a fundamental role in ensuring long-term adherence and improving quality of life [35,36,37]. Dimensions such as vitality, physical pain, perceived health, and psychological well-being appear significantly affected, suggesting the need for a multidimensional care model that integrates therapeutic, rehabilitative, and psychosocial interventions.

The data collected reveal a perceived lack of ongoing care and support, highlighting the urgent need to strengthen the role of nursing professionals within care pathways, especially during the post-diagnostic phase, which is often experienced with disorientation and a sense of isolation. Nonetheless, the study also reveals the emergence of coping strategies among patients, supported by personal resilience and social support.

It would be advisable to expand future research by including a more heterogeneous sample and integrating qualitative tools, such as interviews or follow-up assessments, to further explore the subjective experiences of individuals. Additionally, the development of screening programs aimed at the early detection of suspicious symptoms could help reduce diagnostic delays and improve therapeutic outcomes.

## Figures and Tables

**Table 1:** Socio-demographic characteristics of the sample.

Characteristics	n (%)
**Gender**	
Female	79 (79.8)
Male	20 (20.2)
**Civil status**	
Single	34 (34.3)
Divorced/separated/	11 (11.1)
married	(52.5)
Widowed	2 (2.0)
**Educational level**	
High school diploma	51 (51.5)
Postgraduate training	2 (2.0)
Degree	33 (33.3)
Junior high school degree	13 (13.1)
**Work activity**	
Worker	86 (86.9)
Unemployed	13 (13.1)
**Disease phase**	
Early phase	66 (66.7)
Advanced phase	33 (33.3)
**Multiple Sclerosis typology**	
Primarily progressive multiple sclerosis (MS-PP)	8 (8.1)
Relapsing–remitting multiple sclerosis (MS-RR)	78 (78.8)
Secondarily progressive multiple sclerosis (MS-SP)	7 (7.1)
Clinically isolated syndrome (CIS)	6 (6.1)
**Other family members suffering from MS**	
Yes	24 (24.2)
No	75 (75.8)

**Table 2:** Quality of life perceptions according to MS phase.

Categories Influenced by MS	MS Phase		*p*-Value
	Advanced	Early	
**Relationships with friends and family**			0.644
Very	6 (6.1)	10 (10.1)	
Enough	14 (14.1)	21 (21.2)	
Little	7 (7.1)	19 (19.2)	
None	6 (6.1)	16 (16.2)	
**Relationships with work related colleagues**			0.020 *
Very	8 (8.1)	11 (11.1)	
Enough	2 (2.0)	23 (23.2)	
Little	14 (14.1)	21 (21.2)	
None	9 (9.1)	11 (11.1)	
**Relationship with partner**			0.753
Very	8 (8.1)	18 (18.2)	
Enough	11 (11.1)	19 (19.2)	
Little	5 (5.1)	15 (15.2)	
None	9 (9.1)	14 (14.1)	
**Time devoted to myself**			0.0503
Very	10 (10.1)	20 (20.2)	
Enough	16 (16.2)	26 (26.3)	
Little	5 (5.1)	9 (9.1)	
None	2 (2.0)	11 (11.1)	
**Time devoted to other family members**			0.443
Very	10 (10.1)	16 (16.2)	
Enough	11 (11.1)	17 (17.2)	
Little	8 (8.1)	16 (16.2)	
None	4 (4.0)	17 (17.2)	
**My commitment in the professional sphere**			0.019 *
Very	16 (16.2)	19 (19.2)	
Enough	6 (6.1)	14 (14.1)	
Little	3 (3.0)	24 (24.2)	
None	8 (8.1)	9 (9.1)	
**Daily difficulties encountered**			
**Living with the outcomes of the disease**			0.741
Very	6 (6.1)	11 (11.1)	
Enough	6 (6.1)	10 (10.1)	
Little	4 (4.0)	14 (14.1)	
None	17 (17.2)	31 (31.3)	
**Living with a sense of inadequacy**			0.388
Very	11 (11.1)	13 (13.1)	
Enough	13 (13.1)	27 (27.3)	
Little	7 (7.1)	17 (17.2)	
None	2 (2.0)	9 (9.1)	
**Living with the lack of/absence of assistance**			0.480
Very	10 (10.1)	13 (13.1)	
Enough	12 (12.1)	27 (27.3)	
Little	8 (8.1)	14 (14.1)	
None	3 (3.0)	12 (12.1)	
**Living with uncertainty for the future**			0.046 *
Very	9 (9.1)	5 (5.1)	
Enough	7 (7.1)	12 (12.1)	
Little	10 (10.1)	26 (26.3)	
None	7 (7.1)	23 (23.2)	
**Living with the lack of support from institutions, families, and health care providers**			0.108
Very	20 (20.2)	41 (41.4)	
Enough	3 (3.0)	14 (14.1)	
Little	9 (9.1)	7 (7.1)	
None	1 (1.0)	4 (4.0)	
**Living with an often-inappropriate lifestyle**			0.161
Very	7 (7.1)	13 (13.1)	
Enough	14 (14.1)	18 (18.2)	
Little	10 (10.1)	20 (20.2)	
None	2 (2.0)	15 (15.2)	
**Living with the sexual dysfunction that the disease has caused**			0.030 *
Very	13 (13.1)	14 (14.1)	
Enough	12(2.1)	16 (16.2)	
Little	5 (5.1)	16 (16.2)	
None	3 (3.0)	20 (20.2)	
**Indicate which of these aspects alter the sexual sphere**			
**Loss of desire**			0.003 *
Very	7 (7.1)	2 (2.0)	
Enough	8 (8.1)	7 (7.1)	
Little	8 (8.1)	20 (20.2)	
None	10 (10.1)	37 (37.4)	
**Alterations in sensitivity**			0.688
Very	6 (6.1)	7 (7.1)	
Enough	4 (4.0)	12 (12.1)	
Little	11 (11.1)	23 (23.2)	
None	12 (12.1)	24 (24.2)	
**Lower confidence at the sexual level**			0.303
Very	7 (7.1)	9 (9.1)	
Enough	6 (6.1)	9 (9.1)	
Little	11 (11.1)	17 (17.2)	
None	9 (9.1)	31 (31.3)	
**Feeling less attractive**			0.902
Very	5 (5.1)	7 (7.1)	
Enough	11 (11.1)	21 (21.2)	
Little	8 (8.1)	19 (19.2)	
None	9 (9.1)	19 (19.2)	
**Paresthesias and/or erection problems**			0.772
Very	5 (5.1)	14 (14.1)	
Enough	12 (12.1)	18 (18.2)	
Little	10 (10.1)	20 (20.2)	
None	6 (6.1)	14 (14.1)	
**Decreased vaginal lubrication**			0.028 *
Very	5 (5.1)	3 (3.0)	
Enough	5 (5.1)	10 (10.1)	
Little	10 (10.1)	9 (9.1)	
None	13 (13.1)	44 (44.4)	
**Interference of many other problems that do not allow thinking about sexuality**			0.311
Very	5 (5.1)	4 (4.0)	
Enough	6 (6.1)	11 (11.1)	
Little	6 (6.1)	21 (21.2)	
None	16 (16.2)	30 (30.3)	
**Bladder or bowel disorders**			0.316
Very	7 (7.1)	7 (7.1)	
Enough	8 (8.1)	20 (20.2)	
Little	8 (8.1)	24 (24.2)	
None	10 (10.1)	15 (15.2)	
**Sense of social isolation**			0.015 *
Very	10 (10.1)	5 (5.1)	
Enough	4 (4.0)	9 (9.1)	
Little	9 (9.1)	16 (16.2)	
None	10 (10.1)	36 (36.4)	
**Poor concentration**			0.641
Very	4 (4.0)	6 (6.1)	
Enough	7 (7.1)	9 (9.1)	
Little	9 (9.1)	17 (17.2)	
None	13 (13.1)	34 (34.3)	
**Role changes and/or conflicts**			0.187
Very	5 (5.1)	7 (7.1)	
Enough	12 (12.1)	18 (18.2)	
Little	5 (5.1)	24 (24.2)	
None	11 (11.1)	17 (17.2)	
**Catheter management**			0.046 *
Very	7 (7.1)	4 (4.0)	
Enough	3 (3.0)	5 (5.1)	
Little	11 (11.1)	16 (16.2)	
None	12 (12.1)	41 (41.4)	
**Socio-cultural expectations and biases that inhibit sexuality**			0.028 *
Very	2 (2.0)	0 (0)	
Enough	1 (1.0)	1 (1.0)	
Little	10 (10.1)	9 (9.1)	
None	20 (20.2)	56 (56.6)	

* *p* < 0.05 is statistically significant.

**Table 3:** Impact of disease stage on different dimensions of life.

Domain	Variable	*p*-Value	Group with Greater Impact
Work and relationships	Relationships with colleagues	0.020	Early stage
	Engagement in professional activities	0.019	Early stage
Daily difficulties	Uncertainty about the future	0.046	Advanced stage
	Sexual dysfunction caused by the disease	0.030	Advanced stage
Sexuality	Loss of sexual desire	0.003	Advanced stage
	Decreased vaginal lubrication	0.028	Advanced stage
Social relationships	Sense of social isolation	0.015	Advanced stage
Health management	Catheter management	0.046	Advanced stage
Socio-cultural dimension	Prejudices and expectations causing sexual inhibition	0.028	Advanced stage

## Data Availability

The data that support the findings of this study are available from the corresponding author upon reasonable request. The data are not publicly available due to privacy.

## References

[1] Dobson R., Giovannoni G. (2019). Multiple sclerosis—A review. Eur. J. Neurol..

[2] Hauser S.L., Cree B.A.C. (2020). Treatment of Multiple Sclerosis: A Review. Am. J. Med..

[3] Marcus R. (2022). What Is Multiple Sclerosis?. JAMA.

[4] Associazione Italiana Sclerosi Multipla (2024). Barometro della Sclerosi Multipla e Patologie Correlate. https://agenda.aism.it/2024/download/Barometro_della_Sclerosi_Multipla_2024.pdf.

[5] Powell B., Mills R., Tennant A., Young C.A., Langdon D. (2024). Stigma and health outcomes in multiple sclerosis: A systematic review. BMC Neurol..

[6] Waldman A., Ness J., Pohl D., Simone I.L., Anlar B., Amato M.P., Ghezzi A. (2016). Pediatric multiple sclerosis. Neurology.

[7] Stampanoni Bassi M., Gilio L., Buttari F., Dolcetti E., Bruno A., Galifi G., Azzolini F., Borrelli A., Mandolesi G., Gentile A. (2024). Preventive exercise and physical rehabilitation promote long-term potentiation-like plasticity expression in patients with multiple sclerosis. Eur. J. Neurol..

[8] Savio M., Kearney H., Giunti G. (2025). Evaluating the evidence behind multidisciplinary roles for a multiple sclerosis unit: A systematic literature review. Mult. Scler. Relat. Disord..

[9] Comi G., Leocani L., Ferini-Strambi L., Radaelli M., Costa G.D., Lanzillo R., Lus G., Bianchi V., Traccis S., Capone F. (2023). Impact of treatment with dimethyl fumarate on sleep quality in patients with relapsing-remitting multiple sclerosis: A multicentre Italian wearable tracker study. Mult. Scler. J.—Exp. Transl. Clin..

[10] Messmer Uccelli M., Traversa S., Ponzio M. (2016). A survey study comparing young adults with MS and healthy controls on self-esteem, self-efficacy, mood and quality of life. J. Neurol. Sci..

[11] Benini S., Pellegrini E., Descovich C., Lugaresi A. (2023). Burden and resources in caregivers of people with multiple sclerosis: A qualitative study. PLoS ONE.

[12] Harirchian M.H., Fatehi F., Sarraf P., Honarvar N.M., Bitarafan S. (2018). Worldwide prevalence of familial multiple sclerosis: A systematic review and meta-analysis. Mult. Scler. Relat. Disord..

[13] Penner I.K., Paul F. (2017). Fatigue as a symptom or comorbidity of neurological diseases. Nat. Rev. Neurol..

[14] Costa D.C., Sá M.J., Calheiros J.M. (2012). The effect of social support on the quality of life of patients with multiple sclerosis. Arq. De Neuropsiquiatr..

[15] Timkova V., Mikula P., Linkova M., Szilasiova J., Nagyova I. (2021). Sexual functioning in patients with multiple sclerosis and its association with social support and self-esteem. Psychol. Health Med..

[16] De Nunzio G., Conte L., Lupo R., Vitale E., Calabrò A., Ercolani  M., Carvello M., Arigliani M., Toraldo D.M., De Benedetto L. (2022). A New Berlin Questionnaire Simplified by Machine Learning Techniques in a Population of Italian Healthcare Workers to Highlight the Suspicion of Obstructive Sleep Apnea. Front Med.

[17] Vitturi B.K., Rahmani A., Dini G., Montecucco A., Debarbieri N., Bandiera P., Ponzio M., Battaglia M.A., Brichetto G., Inglese M. (2023). Work Barriers and Job Adjustments of People with Multiple Sclerosis: A Systematic Review. J. Occup. Rehabil..

[18] Gil-González I., Martín-Rodríguez A., Conrad R., Pérez-San-Gregorio M.Á. (2020). Quality of life in adults with multiple sclerosis: A systematic review. BMJ Open.

[19] Mallucci G., Monti M.C., Ponzio M., Borrelli P., Montomoli C., Bergamaschi R. (2024). Impact of multiple sclerosis comorbidities on quality of life and job activity. Mult. Scler. J..

[20] Kavaliunas A., Danylaite Karrenbauer V., Hillert J. (2021). Socioeconomic consequences of multiple sclerosis—A systematic literature review. Acta Neurol. Scand..

[21] Conte L., Rizzo E., Civino E., Tarantino P., De Nunzio  G., De Matteis E. (2024). Enhancing Breast Cancer Risk Prediction with Machine Learning: Integrating BMI, Smoking Habits, Hormonal Dynamics, and BRCA Gene Mutations—A Game-Changer Compared to Traditional Statistical Models?. Appl. Sci..

[22] Vitale E., Lupo R., Calabrò A., Cornacchia M., Conte L., Marchisio D., Caldararo C., Carvello M., Carriero M.C. (2021). Mapping potential risk factors in developing burnout syndrome between physicians and registered nurses suffering from an aggression in Italian Emergency departments. J. Psychopathol..

[23] Lupo R., Vitale E., Panzanaro L., Lezzi A., Lezzi P., Botti S., Rubbi  I., Carvello M., Calabrò M., Puglia A. (2024). Effects of Long COVID on Psycho-Physical Conditions in the Italian Population: A Statistical and Large Language Model Combined Description. Eur. J. Investig. Health. Psychol. Educ..

[24] Kołtuniuk A., Przestrzelska M., Karnas A., Rosińczuk J. (2020). The Association Between Sexual Disorders and the Quality of Life of Woman Patients with Multiple Sclerosis: Findings of a Prospective, Observational, and Cross-Sectional Survey. Sex. Med..

[25] Rocca M.A., Amato M.P., De Stefano N., Enzinger C., Geurts J.J., Penner I.K., Rovira A., Sumowski J.F., Valsasina P., Filippi M. (2015). Clinical and imaging assessment of cognitive dysfunction in multiple sclerosis. Lancet Neurol.

[26] Margoni M., Preziosa P., Rocca M.A., Filippi M. (2023). Depressive symptoms, anxiety and cognitive impairment: Emerging evidence in multiple sclerosis. Transl. Psychiatry.

[27] Jellinger K.A. (2025). Behavioral disorders in multiple sclerosis: A comprehensive review. J. Neural Transm..

[28] Amodeo I., De Nunzio G., Raffaeli G., Borzani I., Griggio A., Conte L., Macchini F., Condò V., Persico N., Fabietti I. (2021). A maChine and deep Learning Approach to predict pulmoNary hyperteNsIon in newbornS with congenital diaphragmatic Hernia (CLANNISH): Protocol for a retrospective study. PLoS ONE.

[29] Arigliani M., Toraldo D.M., Montevecchi F., Conte L., Galasso L., De Rosa F., Lattante C., Ciavolino E., Arigliani C., Palumbo A. (2020). A new technological advancement of the drug-induced sleep endoscopy (Dise) procedure: The “all in one glance” strategy. Int. J. Environ. Res. Public Health.

[30] Zavoreo I., Gržinčić T., Preksavec M., Madžar T., Bašić Kes V. (2016). Sexual Dysfunction and Incidence of Depression in Multiple Sclerosis Patients. Acta Clin. Croat..

[31] Blundell Jones J., Walsh S., Isaac C. (2017). The Relational Impact of Multiple Sclerosis: An Integrative Review of the Literature Using a Cognitive Analytic Framework. J. Clin. Psychol. Med. Settings.

[32] Witzig-Brändli V., Lange C., Gschwend S., Kohler M. (2022). “I would stress less if I knew that the nurse is taking care of it”: Multiple Sclerosis inpatients’ and health care professionals’ views of their nursing-experience and nursing consultation in rehabilitation—A qualitative study. BMC Nurs..

[33] Rubbi I., Lupo R., Lezzi A., Cremonini V., Carvello M., Caricato M., Conte L., Antonazzo M., Caldararo C., Botti S. (2023). The Social and Professional Image of the Nurse: Results of an Online Snowball Sampling Survey among the General Population in the Post-Pandemic Period. Nurs. Rep..

[34] Ponzio M., Santoni L., Molina M., Tavazzi E., Bergamaschi R. (2024). Economic burden of multiple sclerosis in an Italian cohort of patients on disease-modifying therapy: Analysis of disease cost and its components. J. Neurol..

[35] Rieckmann P., Boyko A., Centonze D., Elovaara I., Giovannoni G., Havrdová E., Hommes O., Kesselring J., Kobelt G., Langdon D. (2015). Achieving patient engagement in multiple sclerosis: A perspective from the multiple sclerosis in the 21st Century Steering Group. Mult. Scler. Relat. Disord..

[36] Caldo D., Bologna S., Conte L., Amin M.S., Anselma L., Basile V., Hossain M.M., Mazzei A., Heritier P., Ferracini R. (2023). Machine Learning Algorithms Distinguish Discrete Digital Emotional Fingerprints for Web Pages Related to Back Pain. Sci. Rep..

[37] Lupo R., Zaminga M., Carriero M.C., Santoro P., Artioli G., Calabrò A., Ilari F., Benedetto A., Caslini M., Clerici M. (2020). Eating Disorders and Related Stigma: Analysis among a Population of Italian Nursing Students. Acta Biomed..

